# Clinical Audit of the Awareness of Safety Guidelines on Lithium Prescribing Within the Acute Hospital- James Cook University Hospital

**DOI:** 10.1192/bjo.2022.485

**Published:** 2022-06-20

**Authors:** Nayeema Shakur, Mubin Tahir, Ramanand Badanapuram, Sagrika Nag

**Affiliations:** Tees, Esk and Wear Valley NHS Foundation Trust, Teeside, United Kingdom

## Abstract

**Aims:**

Lithium is a useful drug and is of particular benefit in patients with chronic mood disorders like bipolar affective disorder and recurrent depression. Lithium requires careful monitoring and dose adjustment for safe use due to its narrow therapeutic index and high potential for toxicity. Monitoring must carry on even when mental health patients taking Lithium are admitted to acute hospital. Therefore, the main aim of this clinical audit was to evaluate the level of awareness of the lithium safety guidance amongst medical staff working within the Acute Hospital, James Cook University Hospital. Ideally 100% of staff should have the appropriate knowledge.

**Methods:**

Questionnaire consisted of 6 items which were derived from key points within the Trust guidelines for Lithium. It was designed to highlight the key points in the document and check the level of awareness of the respondents. Respondents were drawn from James Cook University Hospital and South Tees Liaison Psychiatry team. A total of 25 respondents were included in the study.

**Results:**

96% (24/25) of the respondents were aware that renal and thyroid function should be checked for patients on Lithium. 84% (21/25) were aware of the potential impact of Lithium on Kidney function (eGFR) and 68% (17/25) were aware of signs of Lithium toxicity.

60% (15/25) of acute staff were aware about referring patients with deranged Lithium levels to the Liaison Psychiatry team. 40% (10/25) were aware of the drugs that could potentially increase lithium levels like Diuretics, Non-steroidal anti-inflammatory drugs, ACE (angiotensin converting enzyme) inhibitors. Only 24% (6/25) of acute trust staff were aware about checking lithium levels on admission.

**Conclusion:**

Ideally, a 100% compliance and positive response rate should be achieved as these relate to completion of expected safety checks. Lithium is a potentially high-risk drug with a narrow therapeutic index. Possibility of its acute and chronic side effects, including lithium toxicity, makes it essential to follow safety guidelines on lithium prescribing and hence ensure patient safety.

In view of this, the clinical audit results clearly show that there is significant room for improvement to achieve a 100% positive response rate for awareness of safety guidelines on Lithium prescribing.

Overall, there were an average of 57% positive responses and 42% negative responses for awareness of various aspects of the safety guidelines for Lithium.

A robust action plan which included teaching sessions on creating awareness about lithium monitoring was planned because of this audit.

